# The Effects of Drugs on Behavior Maintained by Social Contact: Role of Monoamines in Social Reinforcement

**DOI:** 10.3389/fnbeh.2021.805139

**Published:** 2022-02-21

**Authors:** Jessica L. Sharp, Mark A. Smith

**Affiliations:** Program in Neuroscience, Department of Psychology, Davidson College, Davidson, NC, United States

**Keywords:** dopamine, preclinical model, sex differences, substance use, substance abuse

## Abstract

Drug use is highly concordant among members of adolescent and young adult peer groups. One potential explanation for this observation is that drugs may increase the reinforcing effects of social contact, leading to greater motivation to establish and maintain contact with other members of the peer group. Several classes of drugs, particularly drugs that increase synaptic dopamine, increase the reinforcing effects of contextual stimuli, but the extent to which these drugs enhance the reinforcing effects of social contact is not known. The purpose of this study was to determine the extent to which drugs that increase synaptic dopamine, norepinephrine, and serotonin enhance the positive reinforcing effects of social contact. To this end, male and female Long-Evans rats were pretreated with acute doses of the selective dopamine reuptake inhibitor, WIN-35,428, the selective norepinephrine reuptake inhibitor, atomoxetine, the selective serotonin reuptake inhibitor, fluoxetine, the non-selective monoamine reuptake inhibitor, cocaine, and the non-selective monoamine releasers *d*-amphetamine and (±)-MDMA. Ten minutes later, the positive reinforcing effects of 30-s access to a same-sex social partner was examined on a progressive ratio schedule of reinforcement. To determine whether the reinforcement-altering effects of these drugs were specific to the social stimulus, the reinforcing effects of a non-social stimulus (30-s access to an athletic sock of similar size and coloring as another rat) was determined in control subjects. WIN-35,428, *d*-amphetamine, and cocaine, but not atomoxetine, fluoxetine, or MDMA, dose-dependently increased breakpoints maintained by a social partner under conditions in which responding maintained by a non-social stimulus was not affected. These data indicate that increases in extracellular dopamine, but not extracellular norepinephrine or serotonin, increases the positive reinforcing effects of social contact in both male and female rats. These data also provide support for the hypothesis that some drugs with high abuse liability increase the motivation to establish and maintain contact with social peers.

## Introduction

One of the strongest predictors of whether an adolescent or emerging adult will use drugs is whether his or her friends use drugs (Bahr et al., 2005; Simons-Morton and Chen, 2006; Tompsett et al., 2013; Barnett et al., 2014). Several factors contribute to the high concordance rate of drug use within peer groups, and these factors generally including self-selection processes (e.g., individuals choose peer groups based on shared attitudes and behaviors regarding drugs) and social-learning processes (e.g., drug use is reinforced by other group members either by social praise or continued access to group activities). One potential factor that has received little research attention is the possibility that some drugs increase the motivation to establish and maintain contact with social peers, thus strengthening the bond between drug-using individuals and their respective peer groups.

Access to a social partner is reinforcing and has positive incentive value (Achterberg et al., 2016; Vanderschuren et al., 2016). For instance, contingent access to a castrated male rat maintains responding in female rats at rates comparable to those maintained by food (Evans et al., 1994). Social contact has higher reinforcing efficacy than many common reinforcers, and rats will choose social interaction over intravenous methamphetamine or heroin (Venniro et al., 2018; Venniro and Shaham, 2020). Rats also develop a conditioned place preference for an environment previously paired with a social partner (Calcagnetti and Schechter, 1992; Douglas et al., 2004; Grotewold et al., 2014; Kummer et al., 2014), and greater conditioning is conferred to a partner-paired environment than an amphetamine-paired environment (Yates et al., 2013). Social contact can also reverse a cocaine-induced conditioned place preference and prevent the reinstatement of a cocaine-induced place preference following extinction (Fritz et al., 2011a,b; El Rawas et al., 2012).

Dopamine, norepinephrine, and serotonin play important roles in social behavior. For instance, dopamine increases social approach and social play in rodents (Trainor, 2011; Manduca et al., 2016) and facilitates pair-bonding in monogamous prairie voles (Liu and Wang, 2003; Aragona et al., 2006). Norepinephrine increases prosocial ultrasonic vocalizations (50 kHz) during social interaction, whereas serotonin facilitates maternal bonding and the establishment of a conditioned place preference induced by a social partner (Dölen et al., 2013). Very little research has examined the role these monoamines play in social reinforcement, but at least one study reported that indirect dopamine agonists increase, whereas indirect norepinephrine agonists decrease, the positive reinforcing effects of social play in male rats (Achterberg et al., 2016). Female rats were not tested in that study, and there was not a non-social control stimulus to determine the specificity of the findings.

The purpose of the present study was to determine how drugs targeting dopamine, norepinephrine, and serotonin influence the positive reinforcing effects of social contact relative to a non-social stimulus in both male and female rats. To this end, subjects were pretreated with acute doses of the selective dopamine reuptake inhibitor, WIN-35,428 [(–)-3β-(4-fluorophenyl) tropan-2β-carboxylic acid methyl ester tartrate], the selective norepinephrine reuptake inhibitor, atomoxetine [(R)-N-methyl-γ-(2-methyl-phenoxy) benzenepropanamine hydrochloride], the selective serotonin reuptake inhibitor, fluoxetine [(±)-N-methyl-γ-[4-(trifluoromethyl) phenoxy]benzenepropanamine hydrochloride, the non-selective monoamine reuptake inhibitor, cocaine [(–)-cocaine hydrochloride], the non-selective monoamine releaser *d-*amphetamine [dextroamphetamine hemisulfate salt], and the non-selective monoamine releaser MDMA [(±)-3,4-methylenedioxymethamphetamine hydrochloride]. Ten minutes later, the positive reinforcing effects of 30-s access to a same-sex social partner were examined on a progressive ratio (PR) schedule of reinforcement. To determine whether the reinforcement-altering effects of these drugs were specific to the social stimulus, the reinforcing effects of a non-social stimulus (30-s access to an athletic sock of similar size and coloring as another rat) were determined in control subjects.

## Materials and Methods

### Animals

The subjects were 16 experimentally naïve, adult, male (*n* = 8) and female (*n* = 8), Long Evans rats (*rattus novegicus)*. Rats were obtained from the vendor (Charles Rivers Laboratories, Raleigh, NC) on postnatal day 49 and individually housed in polycarbonate cages (40 cm wide x 85 cm long x 40 cm high) with environmental enrichment. *Ad libitum* access to water and food (LabDiet5P00—ProLabRMH3000) was given to all rats, except for the brief period of lever-press training (see section “Lever-Press Training”). The animal colony was kept on a 12:12 h light-dark cycle (lights on: 0500), with testing occurring during the light portion of the cycle (0900–1500). An additional 6 rats (3 male and 3 female) served as social partners and received no operant training. All rats were maintained in accordance with *the Guide for the Care and Use of Laboratory Animals* as adopted and promulgated by the United States National Institutes of Health, and all procedures were approved the Davidson College Animal Care and Use Committee.

### Apparatus and Chemicals

Testing occurred in operant conditioning chambers from Med Associates (St. Albans, VT). The relevant components of these chambers were a house light, one response lever, and an attached social compartment. The social compartment was located directly opposite the lever and separated from the main chamber by a guillotine door that could be raised to allow social contact between the two rats. A metal screen was affixed in the opening between the two chambers that permitted visual, auditory, olfactory, and limited tactile contact between the two rats, but prevented each rat from traversing from one compartment to the other (see description in Venniro and Shaham, 2020). A white noise machine was also used throughout training and testing.

Cocaine, *d-*amphetamine, MDMA, and WIN-35,428 were generously supplied by the National Institute on Drug Abuse (Research Triangle Institute, Research Triangle Park, NC, United States). Fluoxetine and atomoxetine were purchased from Sigma-Aldrich (St. Louis, MO, United States) and Tocris Bioscience (Minneapolis, MN, United States), respectively. All drugs were dissolved in sterile saline for injection.

### Lever-Press Training

Rats were food restricted to 90% of their free-feeding weight approximately 1 week after arrival. Each rat was trained to lever press using food reinforcement on a fixed ratio (FR1) schedule of reinforcement in operant conditioning chambers separate from those used during testing. Training sessions lasted 2 h or until 40 reinforcers were obtained, whichever occurred first. Rats completed at least four training sessions, and no rat failed to acquire lever-pressing. Following lever-pressing training, all rats returned to *ad libitum* feeding for the remainder of the study.

### Social Assignment and Partnering

Twelve rats (6 male; 6 female) were assigned as test subjects for the social reinforcement experiments. One day before social reinforcement training, each of these rats was placed in a neutral cage with one social partner of the same sex for 15 min. Rats were monitored throughout partnering and returned to their home cages immediately afterward.

Four rats (2 male; 2 female) were assigned to a control group in which the reinforcing stimulus was a black-and-white athletic sock of similar size and coloring as a young-adult, Long-Evans rat. These four rats were included as a reference comparison group to control for drug effects on non-social components of the reinforcing stimulus (e.g., lever retraction, guillotine door opening, novel black-and-white stimulus).

### Social Reinforcement Training and Testing

Social reinforcement training and testing began 1 week following lever-press training. At the beginning of a session, the house light was illuminated, and a single, active response lever was inserted into the chamber. Rats were initially trained to press on a fixed ratio (FR1) schedule of reinforcement in which a single lever press resulted in retraction of the lever, opening of the guillotine door, and 30-s access to the social partner (or the non-social sock stimulus). During the 30-s social access period, test subjects and their social partners had full visual, auditory, and olfactory contact, and limited tactile contact, with one another through the metal screen. Upon completion of the 30-s social access period, the guillotine door lowered, and the lever was reinserted into the chamber. Rats were trained in this manner for 3 days during 1-hr sessions. Contingencies were then changed to a PR schedule of reinforcement for the remainder of the study in which the ratio value systematically increased following each reinforcer: 1, 3, 6, 9, 12, 17, 24, 32, 42, 56, 73, 95, 124, 161, 208, and 268 (for complete algorithm, see Suto et al., 2002).

**FIGURE 1:**
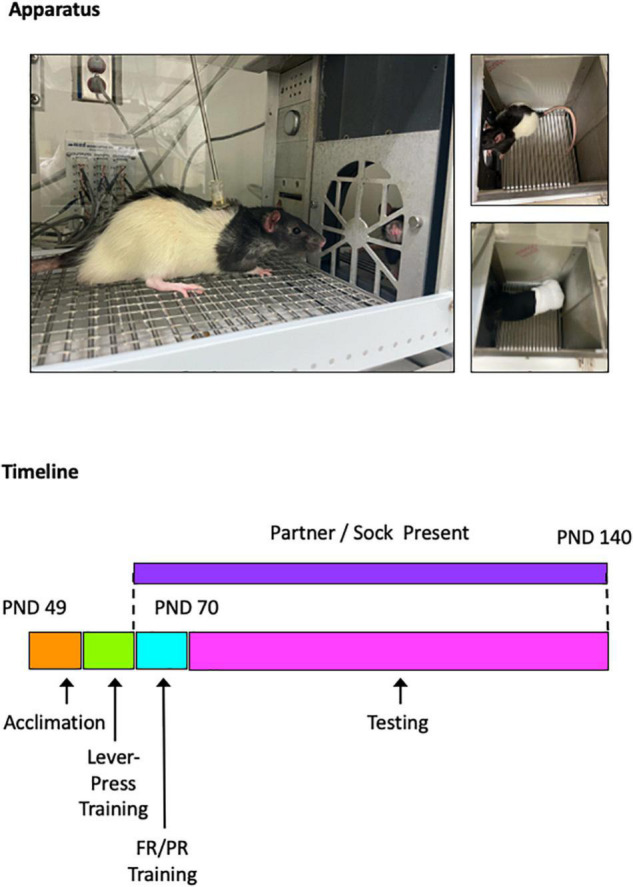
Photographs of experimental apparatus **(top)**. Photographs show experimental subject interacting with a social partner **(left)**, overhead view of social partner **(upper right)**, and overhead view of non-social stimulus (sock; **lower right**). Timeline of experimental events **(bottom)**. Rats were obtained on PND 49, testing began on PND 70, and testing ended on PND 140.

All drug testing was performed during young adulthood, from PND 70 to PND 140. Sessions were conducted 5 days per week, Monday through Friday. Test drugs were administered on Tuesdays and Fridays, and vehicle (saline) was administered on Wednesdays. Training sessions continued Mondays and Thursdays, and data from these sessions are shown as non-injection control sessions. All drugs (and saline) were administered *via* intraperitoneal injection based on body weight 10 min before the test session. The 10-min pretreatment internal was used to ensure (1) peak drug effects occurred during the test sessions and (2) the drugs remained behaviorally active for the full duration of the sessions, which ranged from 90 to 120 min on the PR schedule. Doses for all drugs were administered in a pseudorandomized order with the stipulation that no more than two ascending or descending doses could be tested in a row. Doses were selected based on those shown previously to produce quantifiable effects in other behavioral assays: WIN-35,428 (0.1, 0.3, and 1.0 mg/kg; Kohut et al., 2014), atomoxetine (0.3, 1.0, and 3.0 mg/kg; Choi et al., 2014), fluoxetine (1.0, 3.0, and 10.0 mg/kg; Uzbay et al., 2004), cocaine (1.0, 3.0, and 10.0 mg/kg; Anker et al., 2012), d-amphetamine (0.3, 1.0, and 3.0 mg/kg; Parker et al., 2004), or MDMA (1.0, 3.0, and 10.0 mg/kg; Marti et al., 2019). Depictions of the experimental apparatus and timeline are shown in Figure 1.

### Data Analysis

All 16 rats completed the study. The primary outcome measure was breakpoint, defined as the number of reinforcers (i.e., number of social-access periods) obtained on the PR schedule. Other potential operational definitions of breakpoint (e.g., total number of responses, final ratio value completed) were not used because of violations of sphericity. Data were analyzed *via* mixed-factor ANOVA, with sex as the between-subjects factor and dose as the within-subjects factor. In cases in which a significant main effect of sex or significant sex x dose interaction was observed (3 out of 12 instances), data for males and females were subsequently analyzed separately *via* one-way, repeated-measures ANOVA, with dose serving as the within-subjects factor. In cases in which a main effect of sex or a significant sex x dose interaction was not observed (9 of 12 instances), exploratory analyses were conducted in males and females by the same procedure. In all cases in which an omnibus test was significant, planned pairwise *t*-tests were conducted in which each dose was compared to saline using the Holms-Bonferroni correction for multiple comparisons.

## Results

**FIGURE 2:**
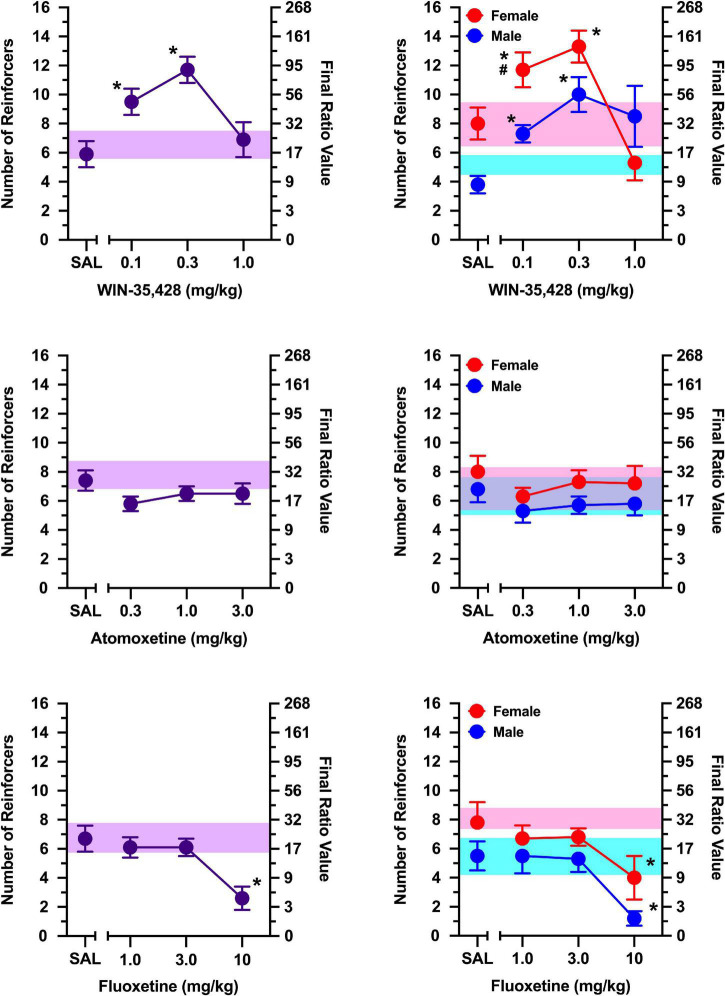
The effects of WIN-35,428 **(top)**, atomoxetine **(middle)**, and fluoxetine **(bottom)** on breakpoints maintained by a social stimulus (i.e., sex- and age-matched partner). Left panels depict data collapsed across males and females. Right panels depict data collected in males and females separately. Left axes reflect breakpoint defined as the number of reinforcers obtained; right panels depict breakpoint as final ratio value completed. Bottom axes represent the effects of saline (SAL) and doses of WIN-35,428, atomoxetine, and fluoxetine expressed in mg/kg. Vertical lines extending from data points represent the SEM. Asterisks (*) indicate significant difference relative to saline. Number sign (^#^) indicates significant difference relative to male rats. Shaded regions indicate variance in breakpoint during non-injection control sessions (purple = collapsed across sex; blue = male; pink = female).

**FIGURE 3:**
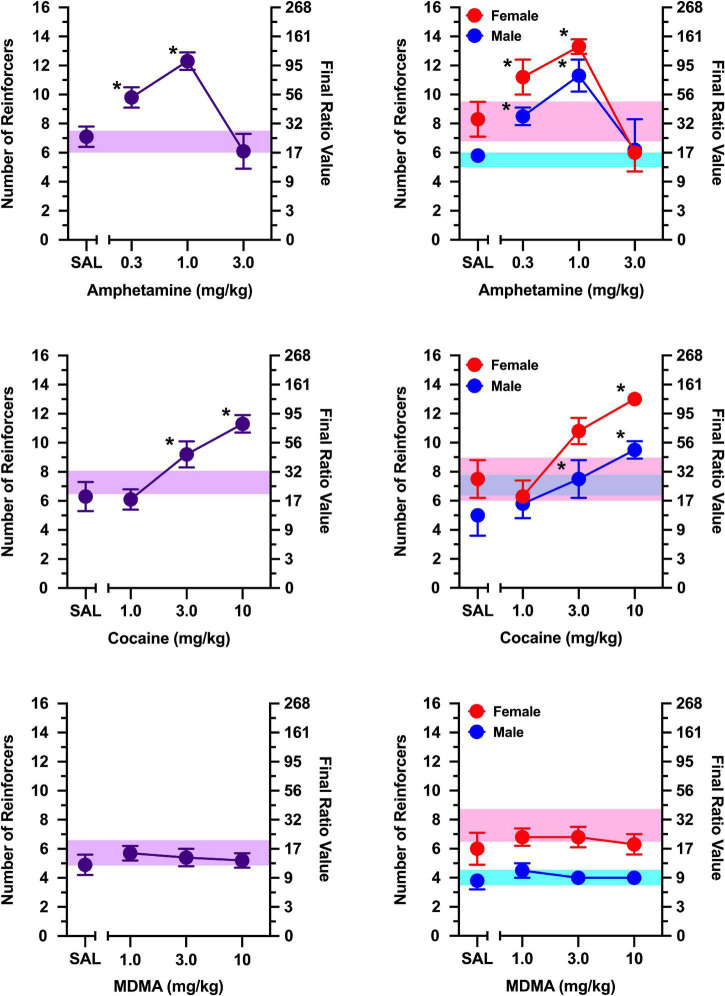
The effects of *d*-amphetamine **(top)**, cocaine **(middle)**, and MDMA **(bottom)** on breakpoints maintained by a social stimulus (i.e., sex- and age-matched partner). Left panels depict data collapsed across males and females. Right panels depict data collected in males and females separately. Left axes reflect breakpoint defined as the number of reinforcers obtained; right panels depict breakpoint as final ratio value completed. Bottom axes represent the effects of saline (SAL) and doses of *d*-amphetamine, cocaine, and MDMA expressed in mg/kg. Asterisks (*) indicate significant difference relative to saline. Shaded regions indicate variance in breakpoint during non-injection control sessions (purple = collapsed across sex; blue = male; pink = female).

**FIGURE 4:**
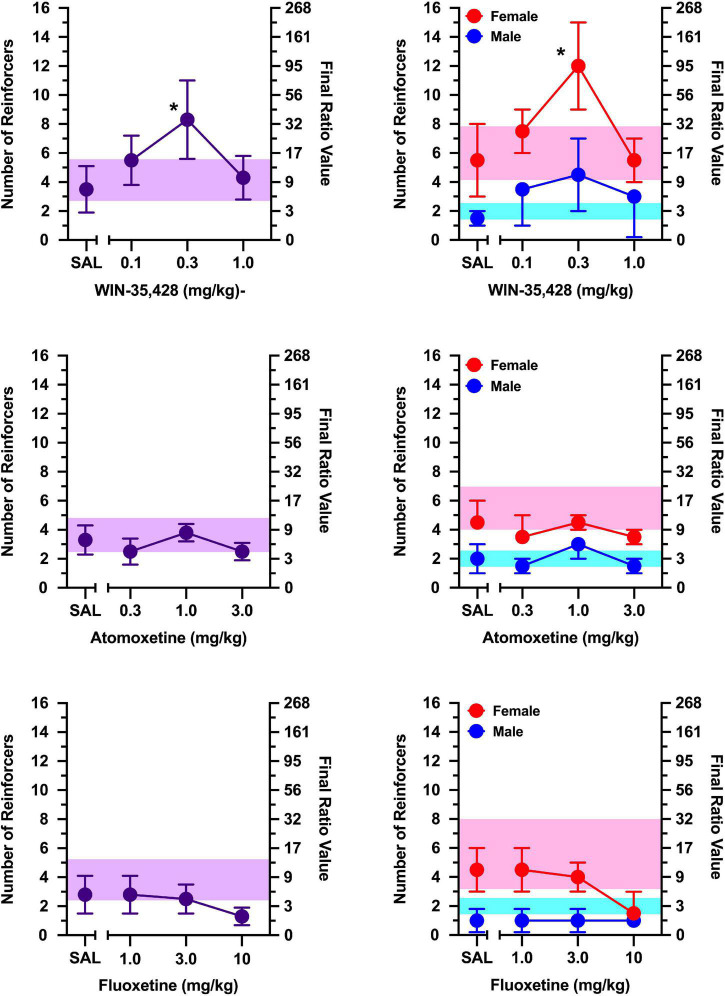
The effects of WIN-35,428 **(top)**, atomoxetine **(middle)**, and fluoxetine **(bottom)** on breakpoints maintained by a non-social stimulus (i.e., black-and-white athletic sock). Left panels depict data collapsed across males and females. Right panels depict data collected in males and females separately. Left axes reflect breakpoint defined as the number of reinforcers obtained; right panels depict breakpoint as final ratio value completed. Bottom axes represent the effects of saline (SAL) and doses of WIN-35,428, atomoxetine, and fluoxetine expressed in mg/kg. Vertical lines extending from data points represent the SEM. Asterisks (*) indicate significant difference relative to saline. Shaded regions indicate variance in breakpoint during non-injection control sessions (purple = collapsed across sex; blue = male; pink = female).

**FIGURE 5:**
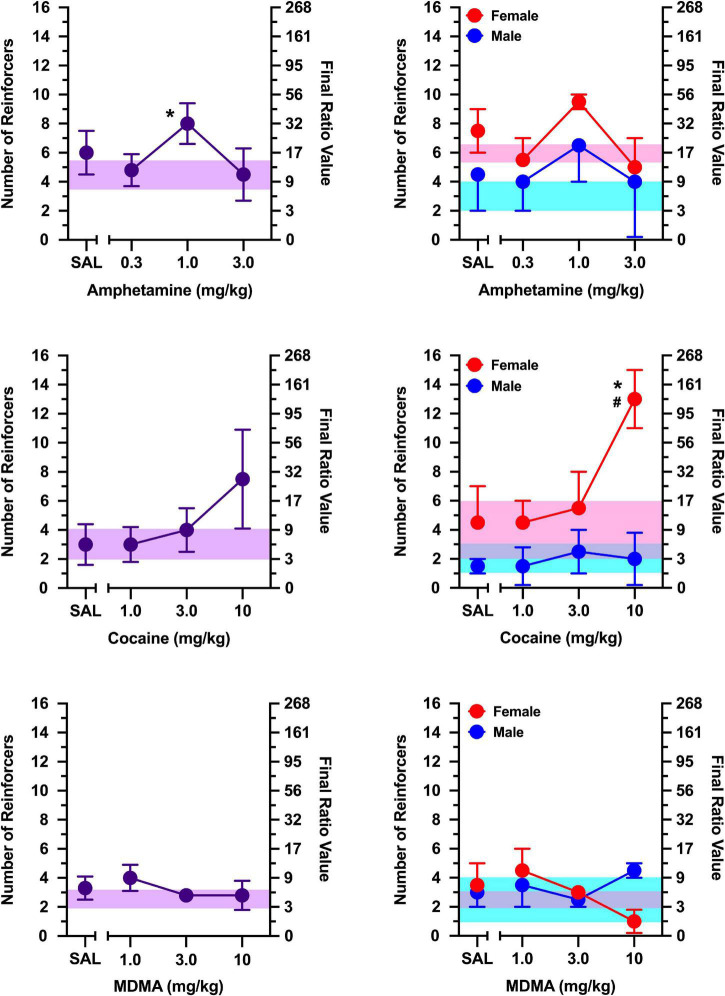
The effects of *d*-amphetamine **(top)**, cocaine **(middle)**, and MDMA **(bottom)** on breakpoints maintained by a non-social stimulus (i.e., black-and-white athletic sock). Left panels depict data collapsed across males and females. Right panels depict data collected in males and females separately. Left axes reflect breakpoint defined as the number of reinforcers obtained; right panels depict breakpoint as final ratio value completed. Bottom axes represent the effects of saline (SAL) and doses of *d*-amphetamine, cocaine, and MDMA expressed in mg/kg. Asterisks (*) indicate significant difference relative to saline. Number sign (^#^) indicates significant difference relative to male rats. Shaded regions indicate variance in breakpoint during non-injection control sessions (purple = collapsed across sex; blue = male; pink = female).

During non-injection control sessions, rats in which responding was maintained by social contact averaged 6–8 reinforcers over the course of the study (Figures 2, 3). In contrast, rats in which responding was maintained by access to a non-social stimulus averaged only 3–5 reinforcers during non-injection control sessions over the course of the study (Figures 4, 5). Females obtained 1–3 reinforcers more than males regardless of the maintaining event.

For rats in which responding was maintained by social contact, a significant sex x dose interaction was observed for the selective dopamine reuptake inhibitor, WIN-35,428 [Figure 2; *F*(3, 30) = 12.968, *p* < 0.001], which was driven by greater responding in females than males at 0.1 mg/kg (*p* = 0.009). WIN-35,428 increased responding maintained by social contact in females [main effect of dose: *F*(3, 15) = 18.959, *p* < 0.001], with 0.1 and 0.3 mg/kg increasing breakpoints relative to saline (*p’*s < 0.001). Similar effects were observed in males; where WIN-35,428 increased responding in a dose-related manner [main effect of dose: *F*(3, 15) = 4.953, *p* = 0.014], with 0.1 mg/kg (*p* = 0.003) and 0.3 mg/kg (*p* = 0.006) increasing breakpoints relative to saline.

The selective norepinephrine reuptake inhibitor, atomoxetine, failed to alter responding maintained by a social stimulus at all doses tested (Figure 2), and this was consistent in both males and females (no main effect of sex, main effect of dose, or sex x dose interaction).

The selective serotonin reuptake inhibitor, fluoxetine, significantly decreased breakpoints maintained by social contact similarly in males and females [Figure 2; main effect of dose: *F*(3, 30) = 11.524, *p* < 0.001], and this was driven by significant decreases in responding at 10 mg/kg relative to saline (*p* < 0.001). Exploratory analyses revealed these effects were apparent in males [main effect of dose: *F*(3, 15) = 7.884, *p* = 0.002] and females [main effect of dose: *F*(3, 15) = 4.265, *p* = 0.023]. A dose of 10 mg/kg decreased responding relative to saline in both males (*p* = 0.020) and females (*p* = 0.034).

WIN-35,428 increased responding maintained by a non-social stimulus [Figure 4; main effect of dose: *F*(3, 6) = 9.721, *p* = 0.010], with 0.3 mg/kg increasing breakpoints relative to saline (*p* = 0.036). The effects of WIN-35,428 did not vary as function of sex (no main effect of sex or sex x dose interaction). An exploratory analysis revealed that WIN-35,428 increased responding by a non-social stimulus in females (main effect of dose: [*F*(3, 3) = 16.704, *p* = 0.022], but this effect was limited to 0.3 mg/kg (*p* = 0.049). WIN-35,428 failed to increase breakpoints maintained by a non-social stimulus in males.

Atomoxetine and fluoxetine failed to alter responding maintained by a non-social stimulus in both males and females at all doses tested (Figure 4; no main effect of sex, main effect of dose, or sex x dose interaction).

The non-selective monoamine releaser, *d-*amphetamine, increased breakpoints maintained by a social stimulus similarly in males and females [Figure 3; main effect of dose: *F*(3, 30) = 19.282, *p* < 0.001], with doses of 0.3 and 1.0 mg/kg increasing responding relative to saline (*p*’s < 0.001). Exploratory analyses revealed these effects were apparent in males [main effect of dose: *F*(3, 15) = 6.143, *p* = 0.006] and females [main effect of dose: *F*(3, 15) = 17.239, *p* < 0.001]. In males, both 0.3 mg/kg (*p* = 0.010) and 1.0 mg/kg (*p* = 0.003) increased responding relative to saline. Similarly, both 0.3 mg/kg (*p* = 0.003) and 1.0 mg/kg (*p* = 0.004) increased responding relative to saline in females.

The non-selective monoamine reuptake inhibitor, cocaine, increased responding maintained by a social stimulus similarly in both males and females [Figure 3; main effect of dose: *F*(3, 30) = 26.628, *p* < 0.001], with 3 mg/kg (*p* = 0.004) and 10 mg/kg (*p* < 0.001) increasing responding relative to saline. Exploratory analyses revealed cocaine increased breakpoints in males [main effect of dose: *F*(3, 15) = 11.805, *p* < 0.001], with doses of 3.0 mg/kg (*p* = 0.037) and 10 mg/kg (*p* = 0.012) increasing responding relative to saline. Cocaine also increased breakpoints in females [main effect of dose: *F*(3, 15) = 15.766, *p* < 0.001], with 10 mg/kg increasing responding relative to saline (*p* = 0.007).

Breakpoints maintained by social contact were greater in females than males during tests with the non-selective monoamine releaser/reuptake inhibitor, MDMA [Figure 3; main effect of sex: *F*(1, 10) = 13.787, *p* = 0.004]. Despite this sex difference, MDMA failed to alter breakpoints in both males and females, with no dose of MDMA increasing or decreasing breakpoints relative to saline in either sex.

*d-*Amphetamine increased responding maintained by a non-social stimulus [Figure 5; main effect of dose: *F*(3, 6) = 8.927, *p* = 0.012], with 1.0 mg/kg increasing responding relative to saline (*p* = 0.016). Although no main effect of sex or sex x dose interaction was observed, exploratory analyses in males and females revealed these effects were driven primarily by females [main effect of dose: *F*(3, 3 = 10.684, *p* = 0.041], but no dose differed significantly from saline.

When responding was maintained by a non-social stimulus, a significant sex x dose interaction was observed for cocaine [Figure 5; *F*(3, 6) = 25.600, *p* < 0.001], which was driven by greater responding in females than males at 10 mg/kg (*p* = 0.030). In females, cocaine increased breakpoints in a dose-dependent manner [main effect of dose: *F*(3, 3) = 73.727, *p* = 0.003], with 10 mg/kg increasing breakpoints relative to saline (*p* = 0.037). In contrast, cocaine failed to increase breakpoints for a non-social stimulus in males.

MDMA did not alter responding maintained by a non-social stimulus under any condition examined (Figure 5).

## Discussion

The purpose of the present study was to determine how drugs targeting dopamine, norepinephrine, and serotonin influence the positive reinforcing effects of social contact in male and female rats while controlling for non-social aspects of the reinforcing stimulus (e.g., lever retraction, guillotine door opening, novel black-and-white stimulus). The principal finding of this study is that drugs with high affinity for the dopamine transporter (DAT) increase the positive reinforcing effects of social contact at a dose (or doses) that do not increase responding maintained by a non-social stimulus. Specifically, at least one dose of WIN-35,428, cocaine, and *d-*amphetamine increased the positive reinforcing effects of social contact at a dose that did not increase responding maintained by a non-social stimulus. In contrast, fluoxetine, atomoxetine, and MDMA did not increase the reinforcing effects of social contact under any of the conditions examined. These findings were consistent across both male and female subjects.

The selective dopamine reuptake inhibitor, WIN-35,428, possesses positive reinforcing effects in laboratory animals (Norman et al., 2004) and increases the reinforcing effects of a conditioned stimulus (Robbins et al., 1983). In the present study, WIN-35,428 increased the positive reinforcing effects of social contact in both male and female rats. A low dose (0.1 mg/kg) selectively increased the reinforcing effects of a social stimulus relative to a non-social stimulus in both sexes, whereas a moderate dose (0.3 mg/kg) selectively increased the reinforcing effects of a social stimulus relative to a non-social stimulus in males only. The dose-effect curve of WIN-35,428 was biphasic, with the highest dose (1.0 mg/kg) failing to increase responding maintained by either stimulus relative to saline-control values. It is not known whether the failure of WIN-35,428 to increase responding at 1.0 mg/kg was due to non-specific motoric effects that interfered with responding (e.g., stereotypies) or the recruitment of other mechanisms that countered its reinforcement-enhancing effects (e.g., anxiety).

The effects observed with WIN-35,428 are consistent with previous studies describing the effects of dopamine on social behavior. In rodents, dopamine increases measures of social play and approach (Trainor, 2011; Manduca et al., 2016). Ultrasonic prosocial vocalizations increase dopamine release in the nucleus accumbens and approaches to a social partner (Willuhn et al., 2014). Dopamine blockade in the lateral septum reduces social play in mice (Bredewold et al., 2018), and dopamine depletion in the prefrontal cortex decreases social interaction in rats (e.g., Espejo, 2003). In monogamous prairie voles, increases in dopamine in the nucleus accumbens facilitate pair bonding (Aragona et al., 2006), whereas blockade of dopamine in the nucleus accumbens prevents the development of a partner preference (Liu and Wang, 2003) and decreases time spent with a familiar partner over an unfamiliar partner (Aragona et al., 2003).

Both the non-selective monoamine reuptake inhibitor, cocaine, and the non-specific monoamine releaser, *d-*amphetamine, have positive reinforcing effects in humans and non-human animals (Porrino et al., 2004; Haney, 2009; Miliano et al., 2016). In the present study, both drugs selectively increased the reinforcing effects of social contact under at least one dose condition. Similar to WIN-35,428, *d-*amphetamine produced a biphasic dose-effect curve, with low and moderate doses (0.3 and 1.0 mg/kg) increasing the reinforcing effects of social contact in both males and females. Importantly, these effects were selective, with neither dose increasing responding maintained by the non-social stimulus. Cocaine increased the reinforcing effects of social contact in a linear fashion, with moderate and high doses (3.0 and 10 mg/kg) significantly increasing responding relative to saline control values. The reinforcement-enhancing effects of 3.0 mg/kg were specific to the social stimulus, whereas the effects of 10 mg/kg were specific to the social stimulus in males only.

In social play situations, cocaine decreases rodent pinning and pouncing but increases social exploration behaviors (e.g., sniffing, grooming; Achterberg et al., 2014). Similarly, *d-*amphetamine increases social approach behaviors (Engelhardt et al., 2017) and prosocial vocalizations (Simola et al., 2018). The effects of cocaine and *d-*amphetamine on the positive reinforcing effects of social contact are likely due to increases in extracellular dopamine. Increases in extracellular dopamine in regions critical to reward and reinforcement (e.g., nucleus accumbens) mediate the positive reinforcing effects of *d-*amphetamine and cocaine in both humans and rodents (Wise, 1984; Koob and Nestler, 1997; España and Jones, 2013), and likely contribute to their ability to increase the reinforcing effects of other stimuli, including social contact (e.g., Tracy et al., 2014; Wright et al., 2018; present study).

Neither the selective norepinephrine reuptake inhibitor, atomoxetine, nor the selective serotonin reuptake inhibitor, fluoxetine, produces positive reinforcing effects in humans or non-human animals (Vanover et al., 1992; Wee and Woolverton, 2004; Gasior et al., 2005). In the present study, neither drug increased the positive reinforcing effects of social contact. Interestingly, a high dose of fluoxetine (10 mg/kg) selectively decreased the reinforcing effects of a social stimulus relative to a non-social stimulus in both males and females. Serotonin increases maternal bonding and is necessary for the establishment of a conditioned place preference for social contact (Dölen et al., 2013), and norepinephrine increases prosocial ultrasonic vocalizations during social interaction (Grant et al., 2018); however, direct and indirect agonists of these two monoamines decrease social play and social approach behavior (Knutson et al., 1996; Homberg et al., 2007; Vanderschuren et al., 2008; Robinson et al., 2012; Achterberg et al., 2015; Houwing et al., 2019). Unlike drugs with high affinity for the DAT, neither serotonin nor norepinephrine reuptake inhibitors increase the reinforcing effects of other stimuli (Sanabria et al., 2008; Higgins et al., 2020).

MDMA is recognized as having abuse liability in humans but functions as only a weak reinforcer in animal models (De La Garza et al., 2007; Schenk et al., 2012). In this study, MDMA failed to increase the positive reinforcing effects of social contact under all conditions examined. This is notable, given MDMA is from the entactogen/empathogen class of drugs, defined by their ability to increase trust, openness, and emotional connection (Nichols, 1986; Dumont and Verkes, 2006; Mithoefer et al., 2016; Sessa, 2018; De Gregorio et al., 2021). In rodent models, MDMA increases adjacent laying in rats, a form of prosocial behavior, and increases social investigation (Morley et al., 2005; Thompson et al., 2009; Ramos et al., 2013); however, MDMA decreases social play (Homberg et al., 2007). MDMA enhances preference for a partner-paired chamber (Kamilar-Britt and Bedi, 2015; Heifets et al., 2019) but also enhances preference for a non-social-stimulus-paired chamber (Ramos et al., 2015). MDMA’s action on serotonin is more pronounced than its action on dopamine (Han and Gu, 2006; Bershad et al., 2016; Wei et al., 2018), and its actions at the serotonin transporter may have masked any reinforcement-enhancing effects that might otherwise be produced by its action at the dopamine transporter.

Collectedly, data obtained with six monoamine releasers and reuptake inhibitors with varying degrees of affinity for the dopamine, norepinephrine, and serotonin transporters indicate that increases in extracellular dopamine play the most prominent role in the positive reinforcing effects of social contact. The same role is not shared by increases in extracellular norepinephrine, given that atomoxetine was devoid of reinforcement-altering effects in this study. Most of the evidence collected in this study suggest that increases in extracellular serotonin decrease the reinforcing effects of social contact and may counter the reinforcement-enhancing effects of dopamine. The effects of all the drugs tested are generally consistent with the effects of monoamines on social play, and the effects of cocaine are consistent with its effects in a previous study examining the positive reinforcing effects of social contact (Achterberg et al., 2016). We emphasize, however, that additional studies with receptor-selective antagonists are needed to determine the receptor mechanisms.

The progressive ratio schedule has advantages over other schedules of reinforcement because it provides a measure of the maximal amount of work (i.e., behavior output) maintained by a stimulus or event (Arnold and Roberts, 1997; Stafford et al., 1998; Roane, 2008). In this sense, it may provide a proxy of extrinsic motivation—a cognitive construct operationally defined as willingness to work for an external reward. This is relevant in the context of how social factors may influence substance use. Specifically, some drugs may strengthen social bonds with individuals immediately present at the time of drug use by increasing the positive reinforcing effects of social contact with those individuals. This process would selectively increase social bonding with individuals in which substance use is viewed as normative.

It is notable that all drugs that increased the positive reinforcing effects of social contact function as robust positive reinforcers in drug self-administration studies. This finding implies that drugs with high abuse liability may be uniquely capable of enhancing the reinforcing effects of social contact and consequently increasing the cohesion of social groups. These data also extend the current literature by demonstrating that the effects of monoamines on social reinforcement are consistent across male and female rats, and by identifying the conditions under which the reinforcement-altering effects of monoamines are specific to a social vs. non-social stimulus.

Some limitations of the study deserve mention. The study used a within-subject design to reduce the number of subjects and to facilitate comparisons across test drugs; it is possible that exposure to multiple drugs coupled with extended training in the same subjects may have affected the results of subsequent behavioral tests. Future studies should replicate these tests using a between-subjects design in experimentally naïve subjects. Although the inclusion of a non-social stimulus condition as a reference comparison group is an advantage, only four subjects were included in that group. Consequently, the control group was underpowered to detect sex differences characterized my small and moderate effect sizes. It is possible that relevant sex differences were missed because of type 2 error in this group. The study also did not include explicit measures of social interaction (e.g., time spent interacting with the partner) or general motor activity (e.g., locomotion). We note that the inclusion of the non-social stimulus control group accounts for general changes in motivated behavior, but future studies would benefit from the inclusion of additional behavioral measures. Finally, other schedules of reinforcement would further characterize how the drugs increase the reinforcing effects of social contact. For instance, concurrent schedules could determine how drugs alter choice between social contact and other reinforcers, whereas conjoint schedules could determine how punishment could attenuate the reinforcement-enhancing effects of these drugs on social contact (Smith, 2020).

Collectively, these findings identify an often-overlooked factor contributing to the high concordance rate of drug use within peer groups. Specifically, some drugs, such as cocaine and other dopaminergic drugs, increase the positive reinforcing effects of social contact, serving to increase social bonds between group members. This factor likely works in conjunction with social factors, such as those involving selection and social learning. According to this hypothesis, an individual may self-select into a group based on shared attitudes and experiences with drugs. Members of that group may then model drug use and reinforce drug use *via* verbal praise or inclusion in the group’s activities. Finally, the use of some drugs increases the reinforcing effects of social contact with other group members, thereby increasing the strength of social bonds within the group, leading to further group conformity and adoption of group norms that encourage drug use.

## Data Availability Statement

The raw data supporting the conclusions of this article will be made available by the authors, without undue reservation.

## Ethics Statement

The animal study was reviewed and approved by the Davidson College Institutional Animal Care and Use Committee.

## Author Contributions

MS developed the research design, secured funding, and contributed to writing the manuscript. JS collected the data, performed the data analysis, and contributed to writing the manuscript. Both authors approved the final draft and are accountable for the work.

## Conflict of Interest

The authors declare that the research was conducted in the absence of any commercial or financial relationships that could be construed as a potential conflict of interest.

## Publisher’s Note

All claims expressed in this article are solely those of the authors and do not necessarily represent those of their affiliated organizations, or those of the publisher, the editors and the reviewers. Any product that may be evaluated in this article, or claim that may be made by its manufacturer, is not guaranteed or endorsed by the publisher.
